# Identification of novel immune-related molecular subtypes and a prognosis model to predict thyroid cancer prognosis and drug resistance

**DOI:** 10.3389/fphar.2023.1130399

**Published:** 2023-03-30

**Authors:** Wei Zhang, Ting Liu, Xinyi Li, Tianshu Li, Xiangchi Ma, Dongxu Zhao, Yueyang Liu, Xueke Zheng, Xudong Zhao

**Affiliations:** ^1^ Department of Endocrinology, Shengjing Hospital of China Medical University, Shenyang, China; ^2^ Department of Otolaryngology-Head and Neck Surgery, Shengjing Hospital of China Medical University, Shenyang, China

**Keywords:** thyroid cancer, immune, prognosis, methylation, molecular subtype, chemotherapy drug

## Abstract

**Background:** Thyroid cancer is a common malignant tumor of the endocrine system that has shown increased incidence in recent decades. We explored the relationship between tumor-infiltrating immune cell classification and the prognosis of thyroid carcinoma.

**Methods:** RNA-seq, SNV, copy number variance (CNV), and methylation data for thyroid cancer were downloaded from the TCGA dataset. ssGSEA was used to calculate pathway scores. Clustering was conducted using ConsensusClusterPlus. Immune infiltration was assessed using ESTIMATE and CIBERSORT. CNV and methylation were determined using GISTIC2 and the KNN algorithm. Immunotherapy was predicted based on TIDE analysis.

**Results:** Three molecular subtypes (Immune-enrich(E), Stromal-enrich(E), and Immune-deprived(D)) were identified based on 15 pathways and the corresponding genes. Samples in Immune-E showed higher immune infiltration, while those in Immune-D showed increased tumor mutation burden (TMB) and mutations in tumor driver genes. Finally, Immune-E showed higher CDH1 methylation, higher progression-free survival (PFS), higher suitability for immunotherapy, and higher sensitivity to small-molecule chemotherapeutic drugs. Additionally, an immune score (IMScore) based on four genes was constructed, in which the low group showed better survival outcome, which was validated in 30 cancers. Compared to the TIDE score, the IMScore showed better predictive ability.

**Conclusion:** This study constructed a prognostic evaluation model and molecular subtype system of immune-related genes to predict the thyroid cancer prognosis of patients. Moreover, the interaction network between immune genes may play a role by affecting the biological function of immune cells in the tumor microenvironment.

## Introduction

Among endocrine tumors, thyroid cancer is a malignant tumor with the highest incidence and manifests low mortality and a relatively favorable prognosis (Wang et al., 2020). However, for locally advanced or recurrent and metastatic thyroid cancer, the existing treatment methods cannot effectively improve patient prognosis. Therefore, novel therapeutic approaches such as immunotherapy targeting the molecular mechanisms of thyroid cancer initiation and progression are under exploration (Farkona et al., 2016). Immune-related genes can be used to predict the prognosis of patients with thyroid cancer and can also serve as therapeutic targets (Gunda et al., 2018). The tumor microenvironment (TME) includes the various cell types (immune cells, fibroblasts, endothelial cells, etc.) and extracellular components (growth factors, cytokines, extracellular matrix, hormones, etc.) surrounding cancer cells (Wu and Dai, 2017). Recent studies have shown that different types of immune cells affect the tumor progression of various cancer types, reflecting TME heterogeneity (Zhang et al., 2020; Chen et al., 2021). Therefore, it is important to understand the role of immune cells and immune genes in the thyroid cancer microenvironment.

Immune checkpoint inhibitors have achieved great efficacy in the treatment of a variety of tumors (Branchoux et al., 2019). In papillary thyroid carcinoma, BRAF V600E mutation is positively correlated with the expression of programmed death ligand 1/programmed death receptor 1 in tumor tissues and immune checkpoint inhibitors can effectively kill thyroid tumor cells (Bai et al., 2018). Gnjatic et al. (2017) found that the number and distribution of tumor-infiltrating immune cells (TIICs) could affect the treatment response in patients with cancer and that TIICs are a potential drug target to further improve patient survival. Inflammation and immune cell infiltration are closely involved in thyroid cancer initiation and development; therefore, the exploration of immune infiltration patterns is needed to evaluate patient treatment response and prognosis (Mould et al., 2017). Immune genes as prognostic molecular markers and potential targets for thyroid cancer immunotherapy have attracted attention (Ma et al., 2020; Zhi et al., 2020; Qin et al., 2021). The current AJCC TNM staging and risk stratification of recurrence for patients with differentiated thyroid cancer are used to guide individualized treatment and are formulated based on the clinicopathological data on patients with thyroid cancer without molecular detection. As the AJCC TNM system is still not sufficiently accurate to classify patients with cancer with different prognoses, patients must be classified at the RNA level.

This study applied bioinformatic methods to identify immune molecular subtypes and construct prognostic models and risk-scoring systems. We evaluated the prognosis of thyroid cancer at the gene and molecular levels and further analyzed the immune gene regulatory network of thyroid cancer to provide new ideas for the study of the immune-related mechanisms of thyroid cancer and the development of immune-targeted drugs.

## Materials and methods

### Raw dataset

RNA-seq, clinical data, transcriptome data, SNV, CNV, and methylation data on patients with thyroid cancer were downloaded from The Cancer Genome Atlas on 23 April 2022. For RNA-seq data, samples without clinical follow-up information, survival time, or status were removed; Ensembl was converted into gene symbol, and the median expression of multiple GeneSymbols was used. The pathological types of thyroid cancer are shown in Table 1.

**TABLE 1:** Pathological types of thyroid cancer.

Var1	Freq
Other, specify	9
Thyroid papillary carcinoma—classical/usual	355
Thyroid papillary carcinoma—follicular (≥99% follicular patterned)	101
Thyroid papillary carcinoma—tall cell (≥50% tall cell features)	36

Data on a total of 15 pathways (immune, stromal, DNA damage repair, and oncogenic) and their corresponding genes were obtained from a previous study (Li and Wang, 2021).

### ssGSEA

ssGSEA analysis was used to calculate the scores of the 15 pathways, EMT pathways (HALLMARK_EPITHELIAL_MESENCHYMAL_TRANSITION), and cytolytic activity (Rooney et al., 2015) using the R package “GSVA”.

### Clustering analysis

Molecular subtyping was performed separately for the TCGA dataset samples *via* ConsensusClusterPlus 1.52.0 using the scores for the 15 pathways (Wilkerson and Hayes, 2010). A total of 500 bootstraps were completed with “pam” arithmetic and “pearson” distances. Each bootstrap involved TCGA dataset specimens (≥80%). The cluster number k was set from 2 to 10, and the optimum k was defined as per cumulative distribution function (CDF) and AUC. Differences in survival (KM) curves were analyzed according to the molecular subtypes. Similarly, the distribution differences in clinical characteristics were compared, and chi-square tests were conducted. *p* < 0.05 was defined as statistically significant. Principal component analysis (PCA) was also performed to test the rationality of the molecular subtype distributions.

### Immune cell infiltration

The proportions of 22 tumor-infiltrating immune cells (TIICs) were calculated using the CIBERSORT algorithm in all malignant tumor samples. ImmuneScore, StromalScore, ESTIMATEScore, and TumorPurity were determined using the ESTIMATE algorithm. ssGSEA identified scores for 28 kinds of immune cells.

### Genetic mutations and epigenetics

For 172 tumor driver genes (159 of which had copy data) (Gao et al., 2013), we used GISTIC2 to analyze the changes in copy number. Those with a ratio >0.2 were considered Gains, while those with a ratio <0.2 were considered Losses; and the rest were considered to be Diploid. SNV was determined using maftools. Methylation of 450K in seven EMT genes and two mismatch repair genes was determined using the KNN algorithm in the impute package.

### Tumor immune dysfunction and exclusion (TIDE)

The TIDE (Jiang et al., 2018; Fu et al., 2020) algorithm (http://tide.dfci.harvard.edu) was used to evaluate the exclusion of CTL and dysfunction of tumor infiltration cytotoxic T lymphocytes (CTL) by immunosuppressive factors.

### Drug sensitivity analysis

The sensitivity to traditional medicines (IC50 values) was predicted using pRRophetic (Geeleher et al., 2014).

### Construction of the IMscore

In the TCGA dataset, thyroid cancer samples were randomly grouped into the training and test datasets in a 1:1 ratio. In the TCGA dataset, we identified pathway genes and pathways with Pearson correlations below the threshold |R| > 0.4, *p* < 0.05 to obtain related genes. In the training dataset, univariate Cox analysis was performed to screen genes related to prognosis. LASSO Cox regression in the glmnet package in R language and stepAIC in the MASS package were performed to select the best prognostic genes. A penalty coefficient *λ* of the optimal value and genes for the model development were determined through 10-fold cross-validation for a total of 1000 iterations. The risk scores for each were calculated using the following formula:

IMscore=∑βi×Expi,

where βi refers to the Cox hazard ratio coefficient of mRNA and Expri is the expression level of a gene. Samples in the training dataset were assigned into two groups of high-risk and low-risk based on the optimal segmentation point cutoff, which was determined using the survminer package. Simultaneously, the effectiveness and robustness of the prognostic risk model were validated in test and entire TCGA datasets. Survival differences among the risk groups were evaluated using Kaplan–Meier (KM) curves combined with log-rank tests. The performance of IMscore in pan-cancer, immunotherapy datasets (IMvigor210 and GSE91061) was also evaluated.

Sangerbox assisted with this article (Shen et al., 2022).

### Statistical analysis

The software packages used in this study were implemented in R software (version 4.2.2; https://www.r-project.org/). A *p*-value < 0.05 was considered statistically significant.

## Results

### Identification of three molecular subtypes in thyroid cancer

**FIGURE 1:**
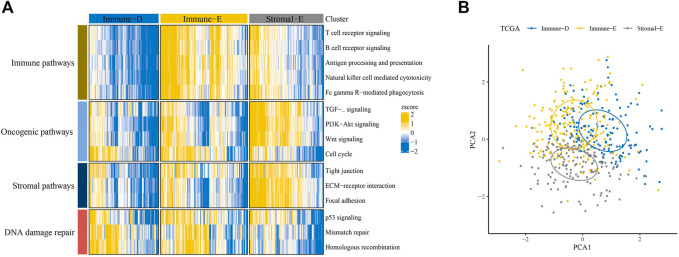
Identification of three molecular subtypes in thyroid cancer. **(A)** Identification of Immune-Enrich, Stromal-Enrich, and Immune-Deprived subtypes in the TCGA dataset. **(B)** Principal component analyses of the three molecular subtypes.

Based on scores in 15 pathways, three molecular subtypes (Immune-Enrich (E), Stromal-Enrich (E), and Immune-Deprived (D)) in thyroid cancer were identified by ConsensusClusterPlus for k = 3 (Figure 1A). The PCA results showed that the three molecular subtypes had distinct boundaries, indicating the rationality of the subtype classification (Figure 1B). Samples in Immune-D showed better OS, while those in Immune-D showed better progression-free survival (PFS) (Supplementary Figure S1A). The distribution of clinical features of the three molecular subtypes indicated the significance of the T and N stages (Supplementary Figure S1B).

### Immune cell infiltration analysis among molecular subtypes

**FIGURE 2:**
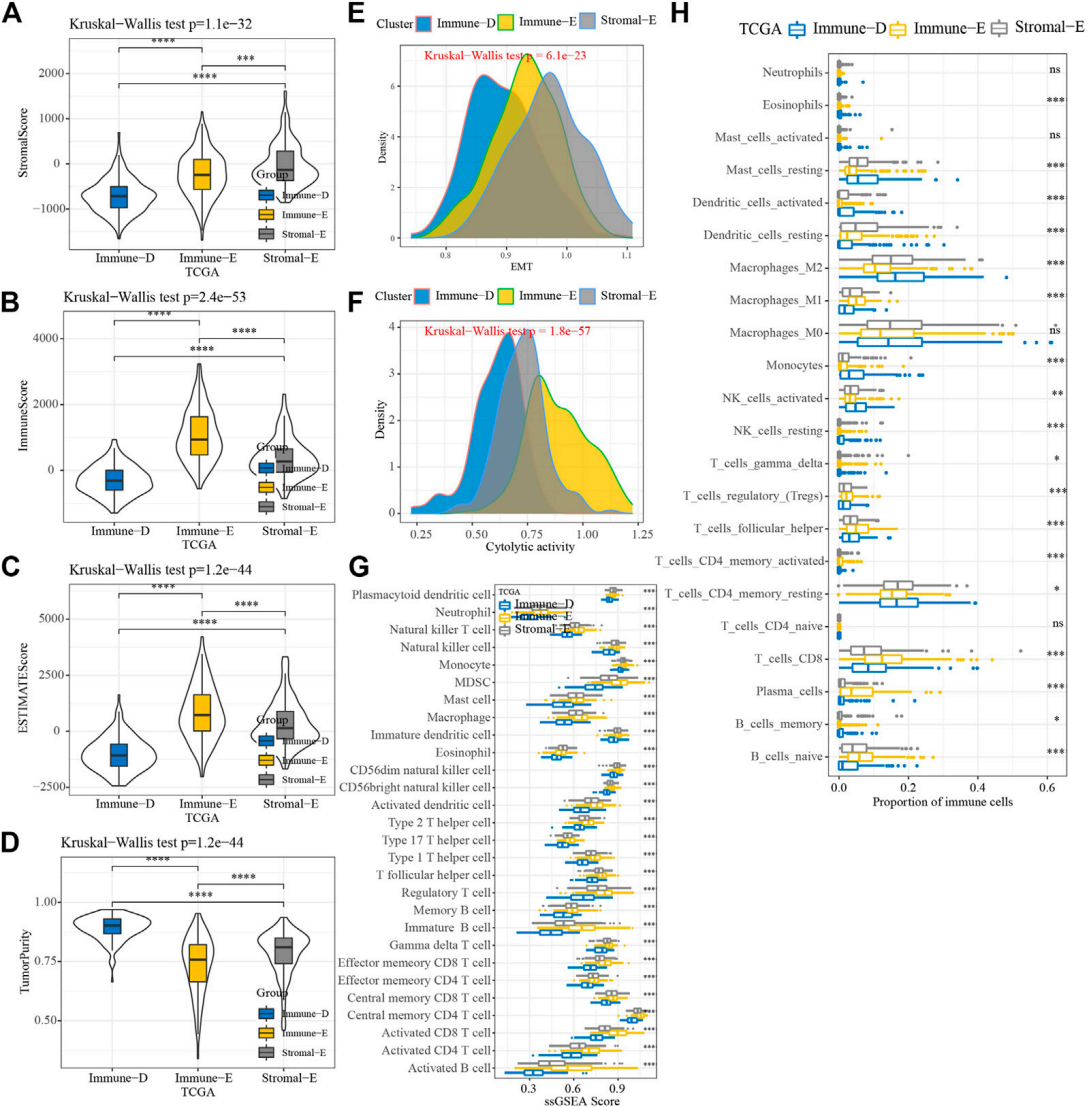
Immune infiltration analysis among three molecular subtypes. **(A)** Distributions of StromalScore among the three molecular subtypes. **(B)** Distributions of ImmuneScore among the three molecular subtypes. **(C)** Distributions of ESTIMATEscores among the three molecular subtypes. **(D)** Distributions of TumorPurity among the three molecular subtypes. **(E)** Differences in EMTscores among the three molecular subtypes. **(F)** Differences in cytolytic activity among the three molecular subtypes. **(G)** Differences in the scores for 28 kinds of immune cells among the three molecular subtypes. **(H)** Differences in the scores for 22 kinds of immune cells among the three molecular subtypes. *p < 0.05; **p < 0.01; ***p < 0.001; ****p < 0.0001; ns: no significance.

The results of the ESTIMATE analysis showed higher and lower ImmuneScore, StromalScore, and ESTIMATEScore in Immune-E and Immune-D, respectively (Figures 2A–C). TumorPurity was lower in Immune-E (Figure 2D). A higher EMT score was observed in Stromal-E (Figure 2E). The cytolytic activity score was increased in Immune-E (Figure 2F). In total, 28 kinds of immune cells showed higher scores in Immune-E (Figure 2G), while 18 of 22 immune cells also had higher scores in Immune-E (Figure 2H).

**FIGURE 3:**
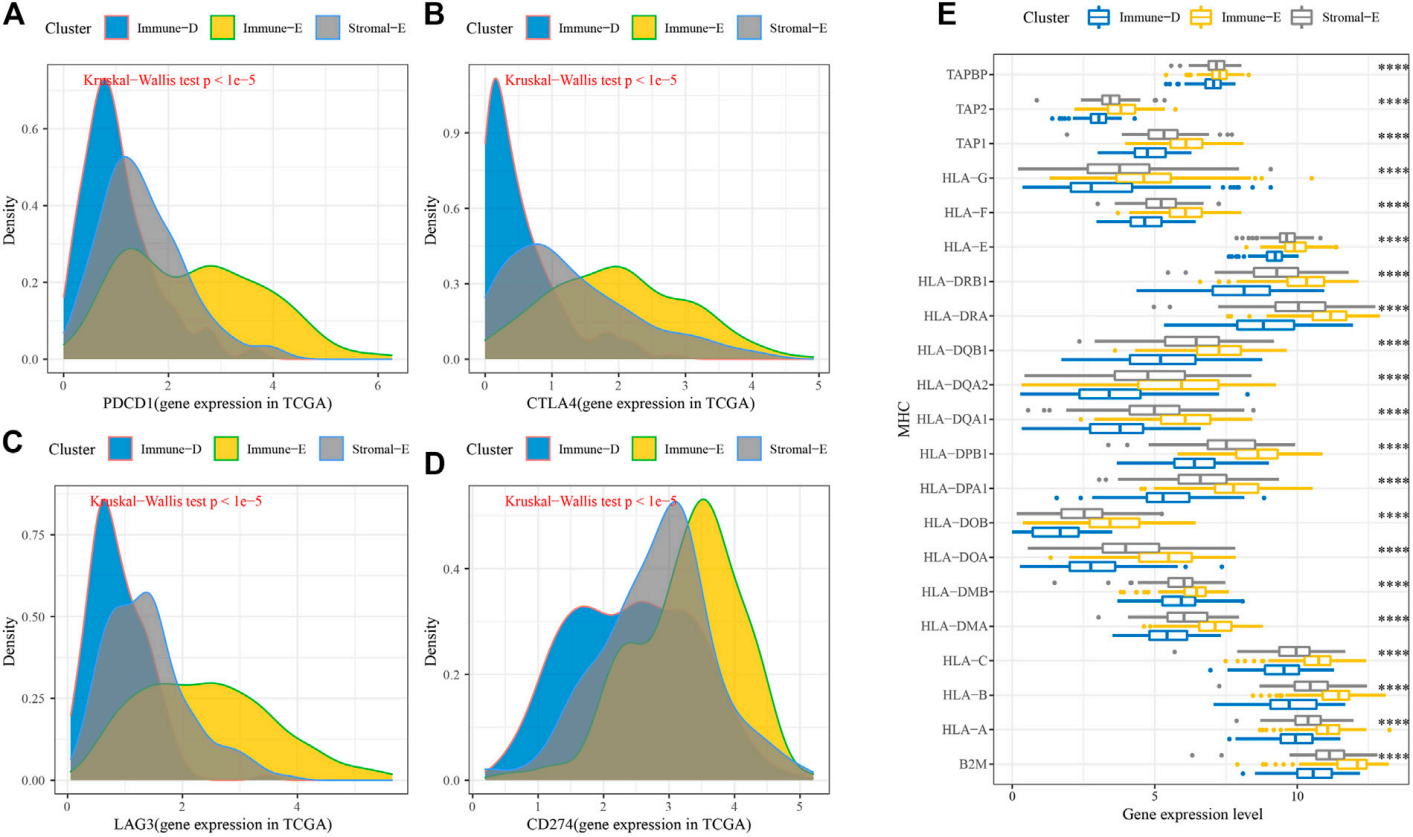
Immune checkpoint genes among the three molecular subtypes. **(A)** Differences in *PDCD1* expression among the three molecular subtypes. **(B)** Differences in *CTLA4* expression among the three molecular subtypes. **(C)** Differences in *LAG3* expression among the three molecular subtypes. **(D)** Differences in *CD274* expression among the three molecular subtypes. **(E)** Differences in MHC-related gene expression among the three molecular subtypes. ****p < 0.0001.

Furthermore, the expression levels of PDCD1, CTLA4, LAG3, and CD274(PD-L1) were upregulated in Immune-E (Figures 3A–D). The expression analysis of MHC genes showed increases in 21 genes in Immune-E (Figure 3E). These results indicated higher immune infiltration in Immune-E.

### Genome mutation analysis of the molecular subtypes

**FIGURE 4:**
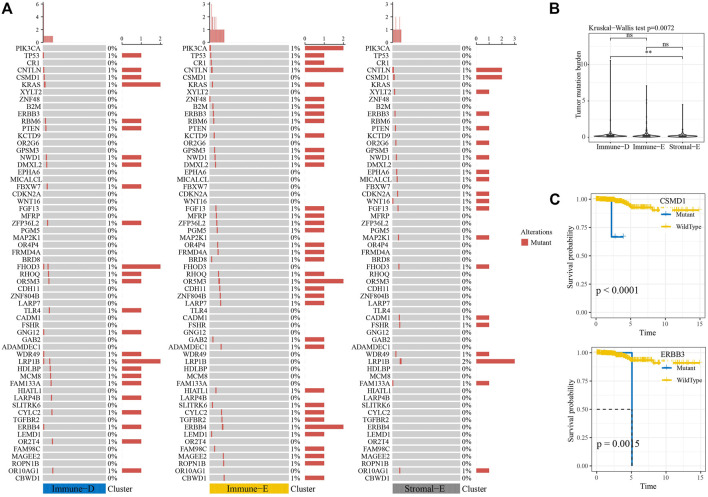
Mutation analysis of tumor-driving genes. **(A)** Mutation analysis of tumor-driving genes among the three molecular subtypes. **(B)** Differences in TBM among the three molecular subtypes. **(C)** KM survival curves of CSMD1 and ERBB3 mutants and wildtype. **p < 0.01; ns: no significance.

**FIGURE 5:**
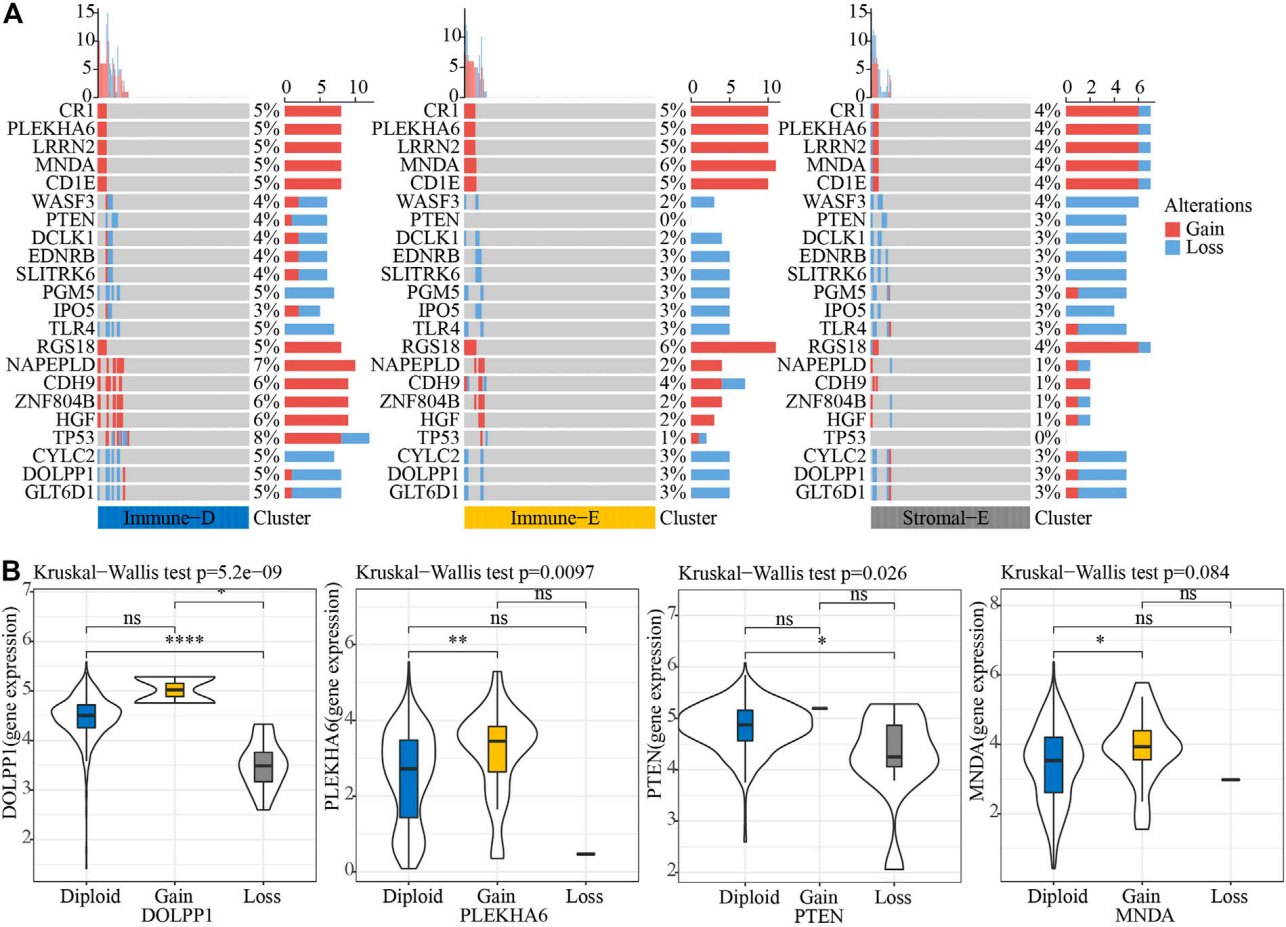
CNV analysis of tumor-driving genes. **(A)** CNV analysis of tumor-driving genes among the three molecular subtypes. **(B)** Expression differences of four genes in three CNV groups. *p < 0.05; **p < 0.01; ****p < 0.0001; ns: no significance.

Next, we analyzed the gene mutations among the molecular subtypes. The results demonstrated that 60 genes among 172 tumor-driving genes showed varying degrees of mutation in the three molecular subtypes (Figure 4A). The TMB was higher in Immune-D compared to Stromal-E (Figure 4B). Tumor driver gene mutations and wild-type samples used for KM analysis showed better survival outcomes in samples with *CSMD1* and *ERBB3* wildtype compared to samples with *CSMD1* and *ERBB3* mutations (Figure 4C). CNV analysis of 159 genes showed copy number amplification and deletion in 22 genes in the three molecular subtypes (Figure 5A). Expression analysis of the corresponding genes in CNV groups of *DOLPP1*, *PLEKHA6*, *PTEN,* and *MNDA* demonstrated that the four genes had higher expression levels in the Gain group and low expression in the Loss group (Figure 5B).

**FIGURE 6:**
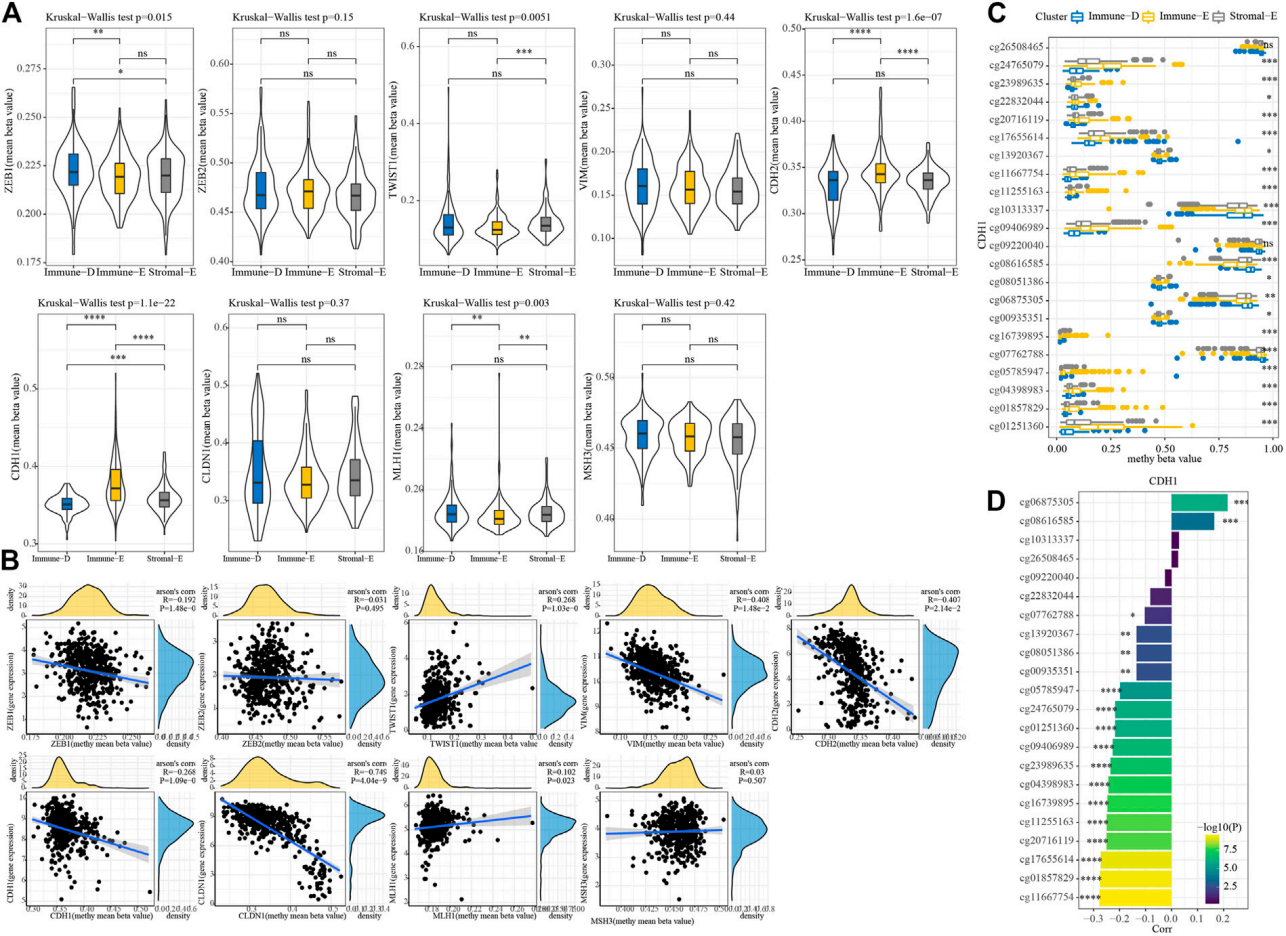
Methylation analysis of genes among the three molecular subtypes. **(A)** 450K beta value differences for nine genes among the three molecular subtypes. **(B)** Correlation analysis between 450K beta values and gene expression. **(C)** Distributions of beta in the cg probe site in *CDH1* among the three molecular subtypes. **(D)** Correlation analysis between 450K beta values of CDH1 and *CDH1* expression. *p < 0.05; **p < 0.01; ***p < 0.001; ****p < 0.0001; ns: no significance.

A total of seven EMT genes and two mismatch repair genes were used to calculate the 450K beta values. The beta values of *ZEB1*, *TW1ST1*, *CDH2*, *CDH1*, and *MLH1* differed among the three molecular subtypes (Figure 6A). Pearson correlation analysis of gene expressions and beta values showed that *ZEB1*, *VIM*, *CDH2*, *CDH1,* and *CLDN1* expressions were negatively correlated with beta value (Figure 6B). The beta value of the cg probe site in *CDH1* was higher in Immune-E (Figure 6C). Similarly, the beta value of the cg probe site was negatively correlated with CDH1 expression (Figure 6D).

### Immunotherapy prediction and drug sensitivity analysis

**FIGURE 7:**
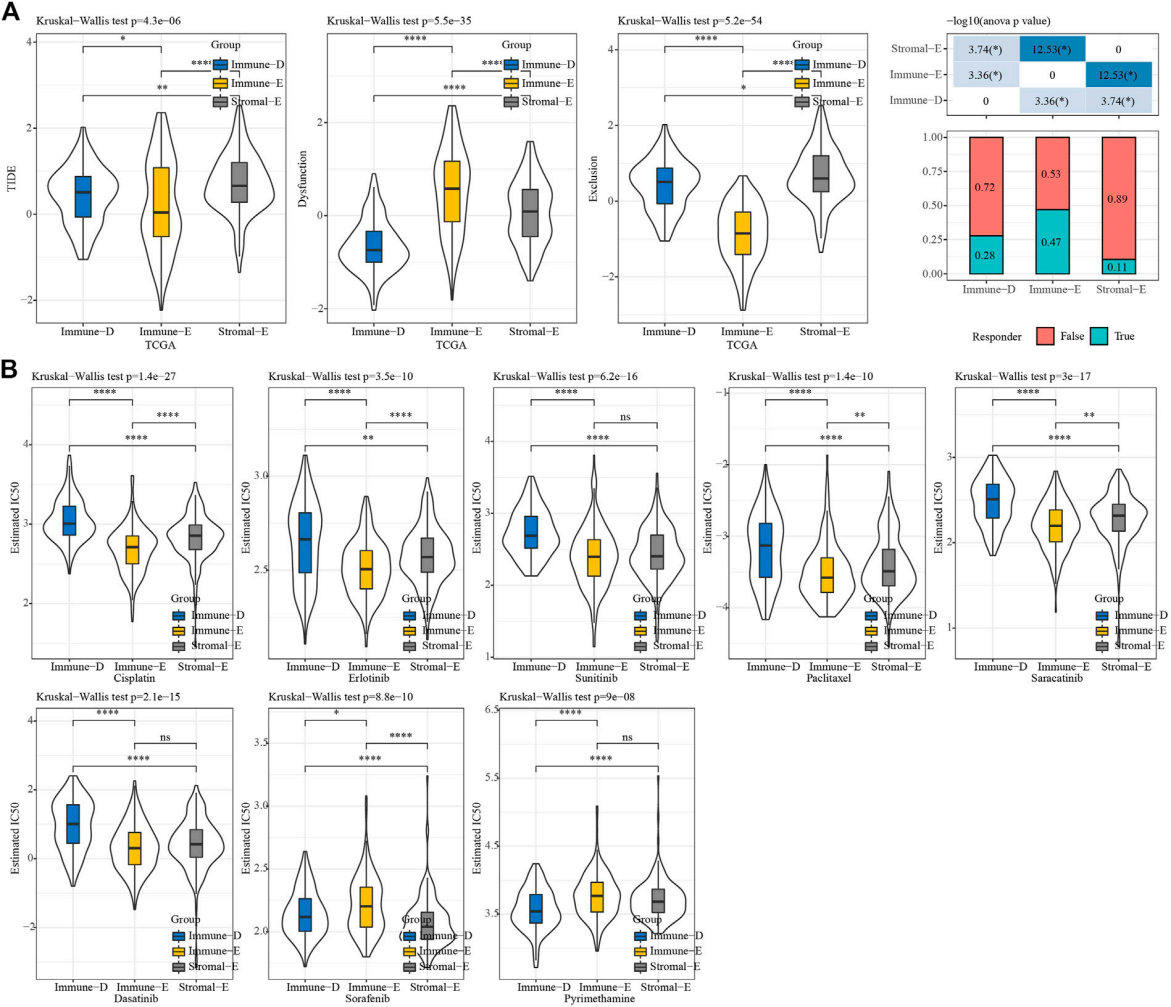
TIDE and drug sensitivity analysis. **(A)** TIDE analysis among the three molecular subtypes. **(B)** IC_50_ analysis of eight drugs among the three molecular subtypes. *p < 0.05; **p < 0.01; ****p < 0.0001; ns: no significance.

We used TIDE (http://tide.dfci.harvard.edu/) software to evaluate the potential clinical effect of immunotherapy according to the molecular subtypes. The TIDE score was lower for Immune-E, indicating that Immune-E may be more suitable for immunotherapy. Moreover, 47% of samples in Immune-E showed immunotherapy response, a proportion higher than those in Stromal-E and Immuno-D (Figure 7A). The IC_50_ values for cisplatin, erlotinib, sunitinib, paclitaxel, saracatinib, and dasatinib were lower in Immune-E, suggesting that Immune-E is more sensitive to those chemotherapeutic drugs (Figure 7B).

### Construction of the IMScore

In the TCGA dataset, Pearson correlation analysis between genes in pathways and pathways identified 1784 genes with |R|>0.4 and *p* < 0.05. Then, in the TCGA training dataset, seven prognosis genes (*p* < 0.05) for thyroid cancer were screened from 1784 genes using univariate Cox analysis. Finally, four genes were used to construct a prognostic model (IMScore = 0.732*HSPA6+0.917*FLNC 1.083*CLDN2 0.966*E2F1) through lasso analysis and the stepAIC method.

**FIGURE 8:**
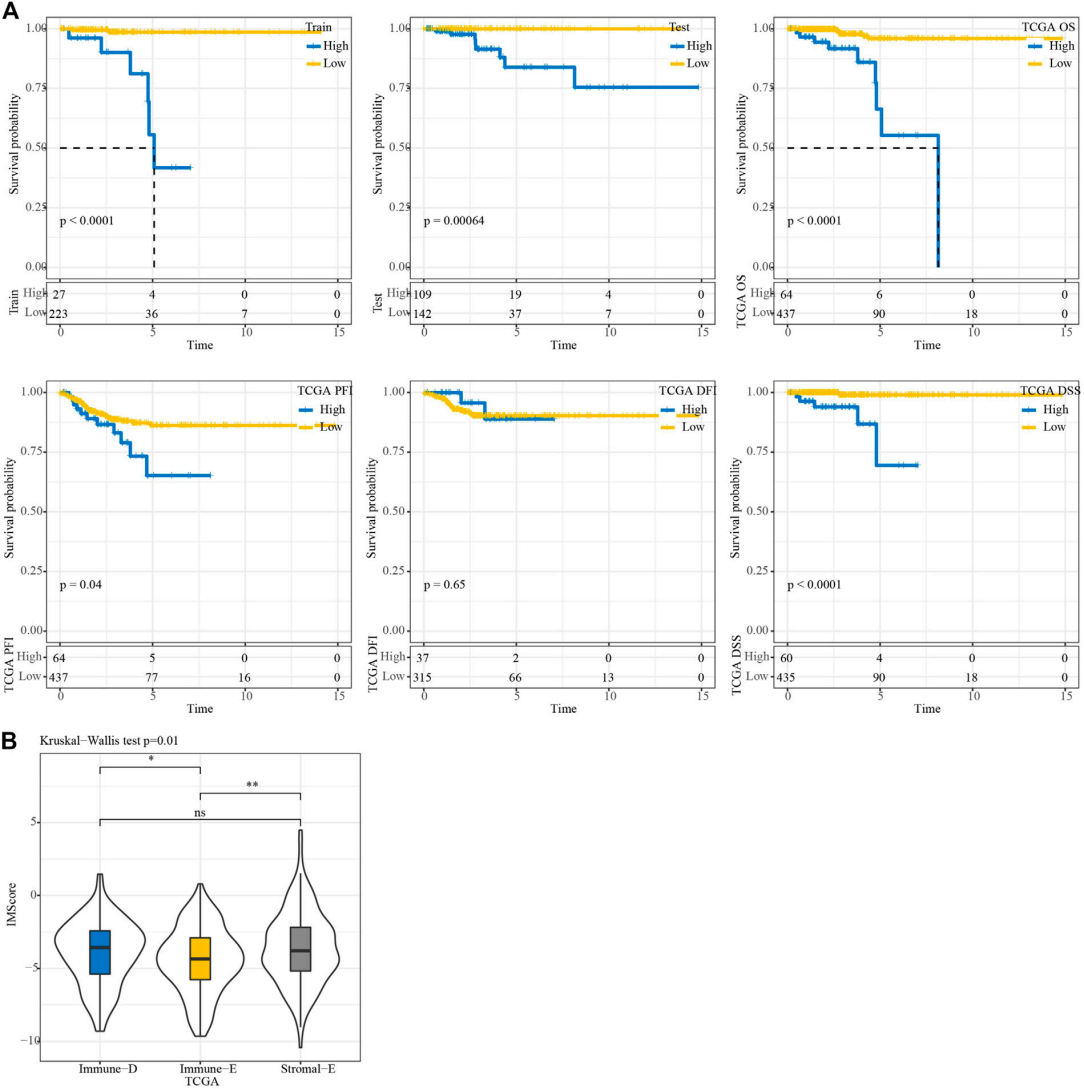
KM survival analysis. **(A)** KM survival analysis of the high and low groups in TCGA train, test, entire, TCGA-PFI, TCGA-DFI, and TCGA-DSS datasets. **(B)** Differences in IMScore among the three molecular subtypes.

In the TCGA training, testing, and entire TCGA datasets, samples were classified into high and low IMScore groups using the cutoff. KM curve analysis showed that patients in the low group had longer survival times. Moreover, in terms of PFS, DFI, and DSS, the low group showed better PFI (*p* = 0.04) and DSS (*p* < 0.0001) (Figure 8A). The IMScores were higher in the Immune-D and Stromal-E subtypes (Figure 8B).

### Performance prediction of the prognostic model

**FIGURE 9:**
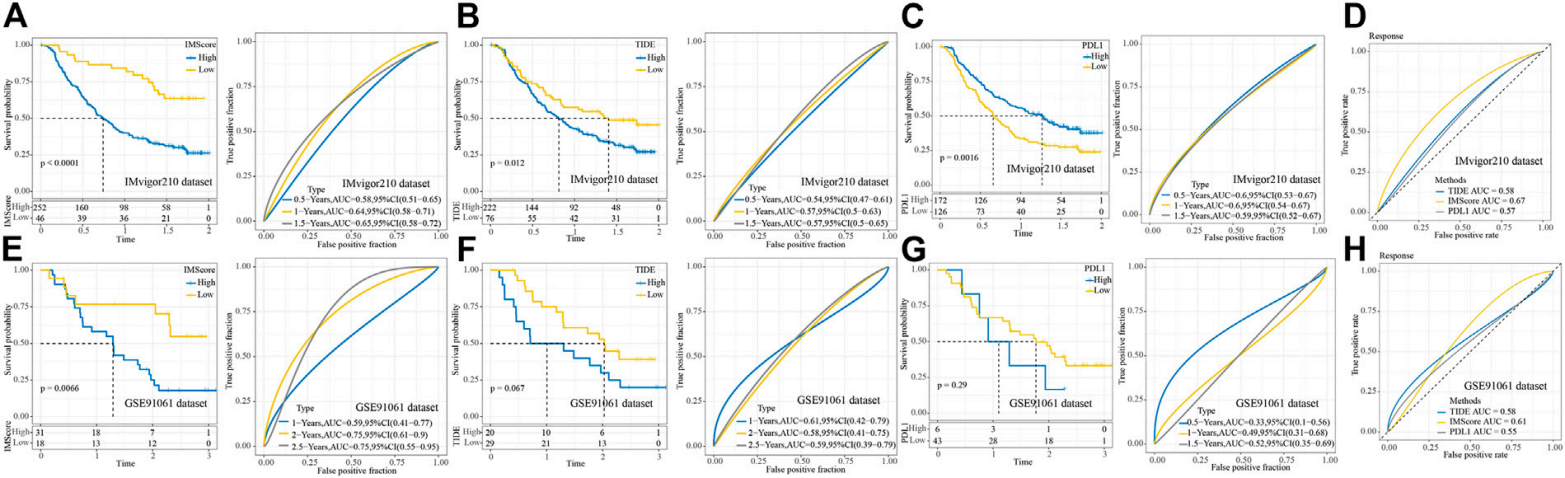
Performance of IMScore in immunotherapy datasets. **(A)** KM survival curve and ROC analysis of IMScore in the IMvigor210 dataset. **(B)** KM survival curve and ROC analysis of TIDE in the IMvigor210 dataset. **(C)** KM survival curve and ROC analysis of PD-L1 in the IMvigor210 dataset. **(D)** ROC analysis of IMScore and TIDE in the IMvigor210 dataset. **(E)** KM survival curve and ROC analysis of IMScore in the GSE91061 dataset. **(F)** KM survival curve and ROC analysis of TIDE in the GSE91061 dataset. **(G)** KM survival curve and ROC analysis of PD-L1 in the GSE91061 dataset. **(H)** ROC analysis of IMScore and TIDE in the GSE91061 dataset.

Among 32 cancer types in the TCGA dataset, high IMScore survival times were shorter than low IMScore survival times except for TGCT and UCS (Supplementary Figure S2). We validated the prediction effect of IMScore in the immunotherapy datasets IMvigor210 and GSE91061. In the IMvigor210 dataset, samples with a low IMScore had better survival outcomes, and the 0.5-, 1-, and 1.5-year AUCs were 0.58, 0.64, and 0.65, respectively (Figure 9A). The samples with low TIDE had better survival outcomes, and the 0.5-, 1-, and 1.5-year AUCs were 0.54, 0.57, and 0.57, respectively (Figure 9B). Samples with low PD-L1 also had better survival outcomes, and the 0.5-, 1-, and 1.5-year AUCs were 0.6, 0.6, and 0.59, respectively (Figure 9C). The prediction of the response to treatment showed AUCs of TIDE, PD-L1, and IMScore of 0.58, 0.57, and 0.67, respectively (Figure 9D). In the GSE91061 dataset, samples with a low IMScore had better survival outcomes, and the 0.5-, 1-, and 1.5-year AUCs were 0.59, 0.75, and 0.75, respectively (Figure 9E). The samples with low TIDE had better survival outcomes, and the 1-, 2-, 2.5-year AUCs were 0.61, 0.58, and 0.59, respectively (Figure 9F). The survival outcomes did not differ significantly between the low- and high- PD-L1 groups, and the 0.5-, 1-, 1.5-year AUCs were 0.54, 0.57, and 0.57, respectively (Figure 9G). The prediction of the response to treatment showed AUCs of TIDE, PD-L1, and IMScore of 0.58, 0.55, and 0.61, respectively (Figure 9H). The results of the aforementioned analyses demonstrated the better prediction effect of the IMScore compared to TIDE.

### Nomogram model of thyroid cancer

**FIGURE 10:**
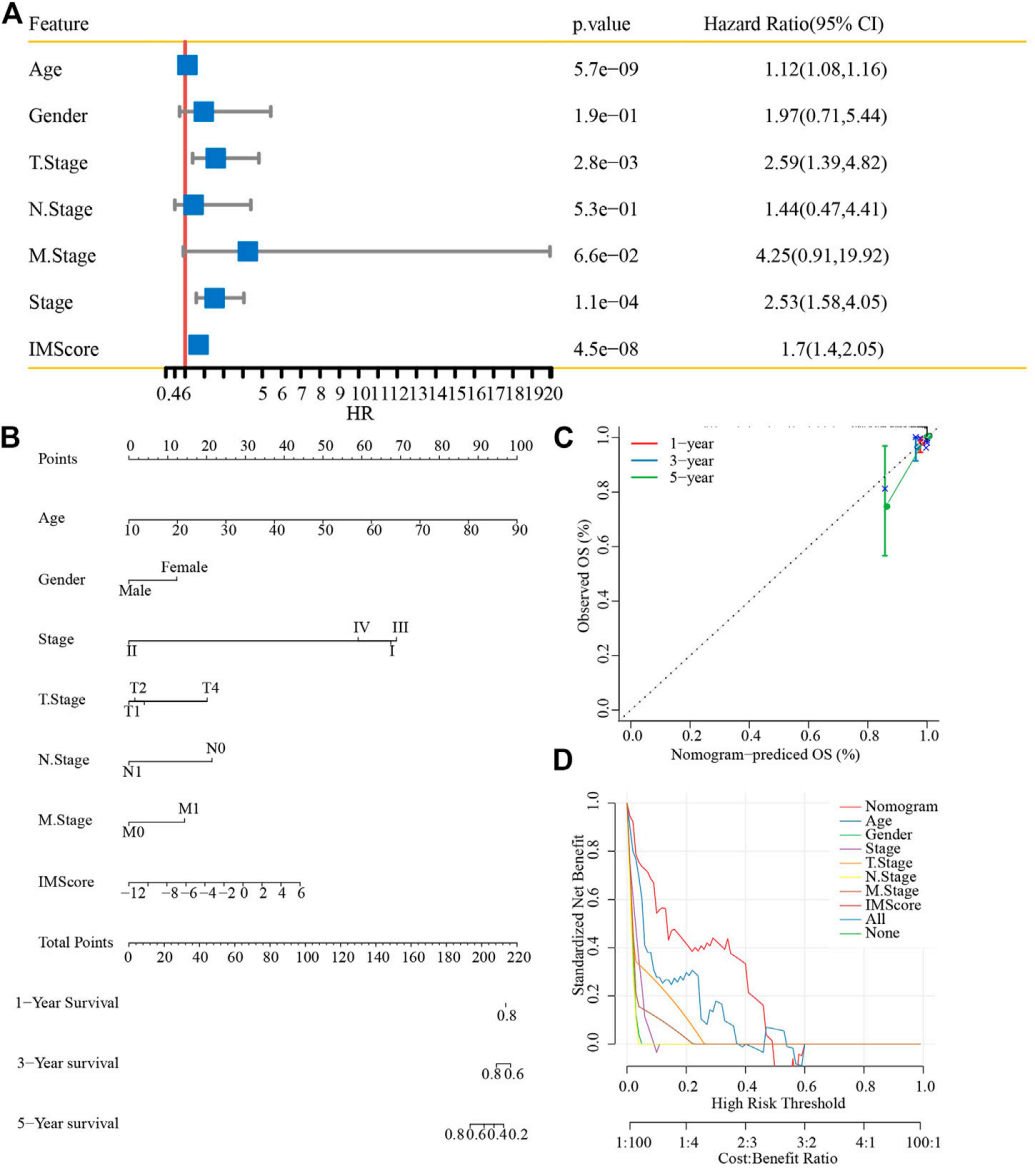
Nomogram construction. **(A)** Univariate analysis of the IMScore and clinical features. **(B)** Nomogram that incorporated the IMScore and clinical features was developed. **(C)** Calibration curve. **(D)** Decision curve analysis.

First, univariate analysis showed that age, gender, TNM stage (*p* < 0.001), stage, and IMScore were significantly associated with a shorter OS in patients with thyroid cancer (Figure 10A). Then, we established a nomogram model that included the important predictors in the Cox analysis to predict the prognosis of thyroid cancer (Figure 10B). The calibration curve showed good concordance between the predicted and observed values of 1-, 3-, and 5-year OS (Figure 10C). The decision curve showed that the nomogram had the best prediction performance for the prognosis of thyroid cancer (Figure 10D).

## Discussion

The main obstacle to tumor progression is the immune system, which sees tumors as emerging pathogens that require elimination (Martin et al., 2021). Understanding tumor immunity is critical for improving current immunotherapy regimens. In 2018, Thorsson et al. (2018) developed a new immune classification system comprising six immune subtypes: C1 (wound healing), C2 (IFN-γ phenotype), C3 (inflammatory), C4 (lymphocyte depletion), C5 Type I (immunosilencing), and type C6 (TGF-β dominant). In different tumors, different immune subtypes have different prognoses, and patients with C4 and C6 tumors have worse prognoses. In colorectal cancer, the immune subtypes are mainly types C1 and C2. Different immune subtypes cause different biological differences, which may explain drug heterogeneity in patients with traditional cytotoxic drugs and immunotherapy (Soldevilla et al., 2019). We divided thyroid cancer samples into three immune types based on immune cells: Immune-enrich (E), Stromal-enrich(E), and Immune-deprived(D). Immuno-E showed a high immune cell infiltration but shorter OS, probably because of a small number of dead samples (7.96%).

Cancer is essentially a genomic disease that progresses as mutations including CNVs and SNPs accumulate in somatic cells, as well as epigenomic alterations with or without inherited alterations. CNV is one of the most common markers in the cancer genome, which can lead to oncogene activation and tumor suppressor gene inactivation (Nakagawa and Fujita, 2018). DNA methylation is the most important epigenetic variation in the human genome, and the process of cell carcinogenesis is always accompanied by extensive changes in DNA methylation (Locke et al., 2019; Pan et al., 2021). To further explore the underlying differences in mechanism among the three immune subtypes, we selected methylation and gene copy number. We detected CNV and hypermethylation of tumor driver genes in all three subtypes. The methylation and copy values of genes were negatively and positively correlated with mRNA expression levels, respectively; hence, the differences between thyroid cancer subtypes may be due to changes in gene copy number and methylation.

In this study, biological information analysis identified four genes, *HSPA6*, *FLNC*, *CLDN2*, and *E2F1* as candidate biomarkers of thyroid cancer. Recently, *HSPA6* was found to be indispensable in the Withaferin A-mediated inhibition of apoptosis/autophagy or migration in breast cancer cells (Hahm et al., 2021). Alterations in Claudin-2 (*CLDN2*), a component of cellular tight junction, are involved in the progression of a variety of cancer types (Buchert et al., 2010; Tabariès et al., 2011; Tabariès et al., 2012). *E2F1* is a potent oncogene in human cancers, including thyroid cancer, prostate cancer, lung cancer, and colorectal cancer, that can accelerate the invasion, spread, and metastasis of cancer cells and further predict poor prognosis (Bi et al., 2017; Yin et al., 2017; Zhou et al., 2020; Yang et al., 2022).

Although we used bioinformatics methods on large numbers of samples to identify genetic subgroups and develop a prognosis model of thyroid carcinoma that showed significant prognostic differences, this study has several limitations. Future work will place a greater emphasis on research that is both fundamentally experimental and functionally in-depth. Moreover, we were unable to consider other factors because the samples lacked essential clinical follow-up information, such as diagnostic specifics; for instance, whether the patients had other health conditions. These factors may have informed the differentiation of the molecular subtypes.

In conclusion, we identified three immune molecular subtypes and developed a prognostic model based on four prognostic genes, which may provide new targets for the diagnosis and treatment of thyroid cancer. Further studies are needed to confirm the mechanism of prognostic genes, which will provide new opportunities for the diagnosis and treatment of thyroid cancer.

## Data Availability

The original contributions presented in the study are included in the article/Supplementary Material. Further inquiries can be directed to the corresponding author.
