# Supplementary figures and images for: Identification of novel immune-related molecular subtypes and a prognosis model to predict thyroid cancer prognosis and drug resistance
Source: Front Pharmacol. 2023 Mar 30;14:1130399. doi: 10.3389/fphar.2023.1130399 (PMC10098004; doi:10.3389/fphar.2023.1130399)

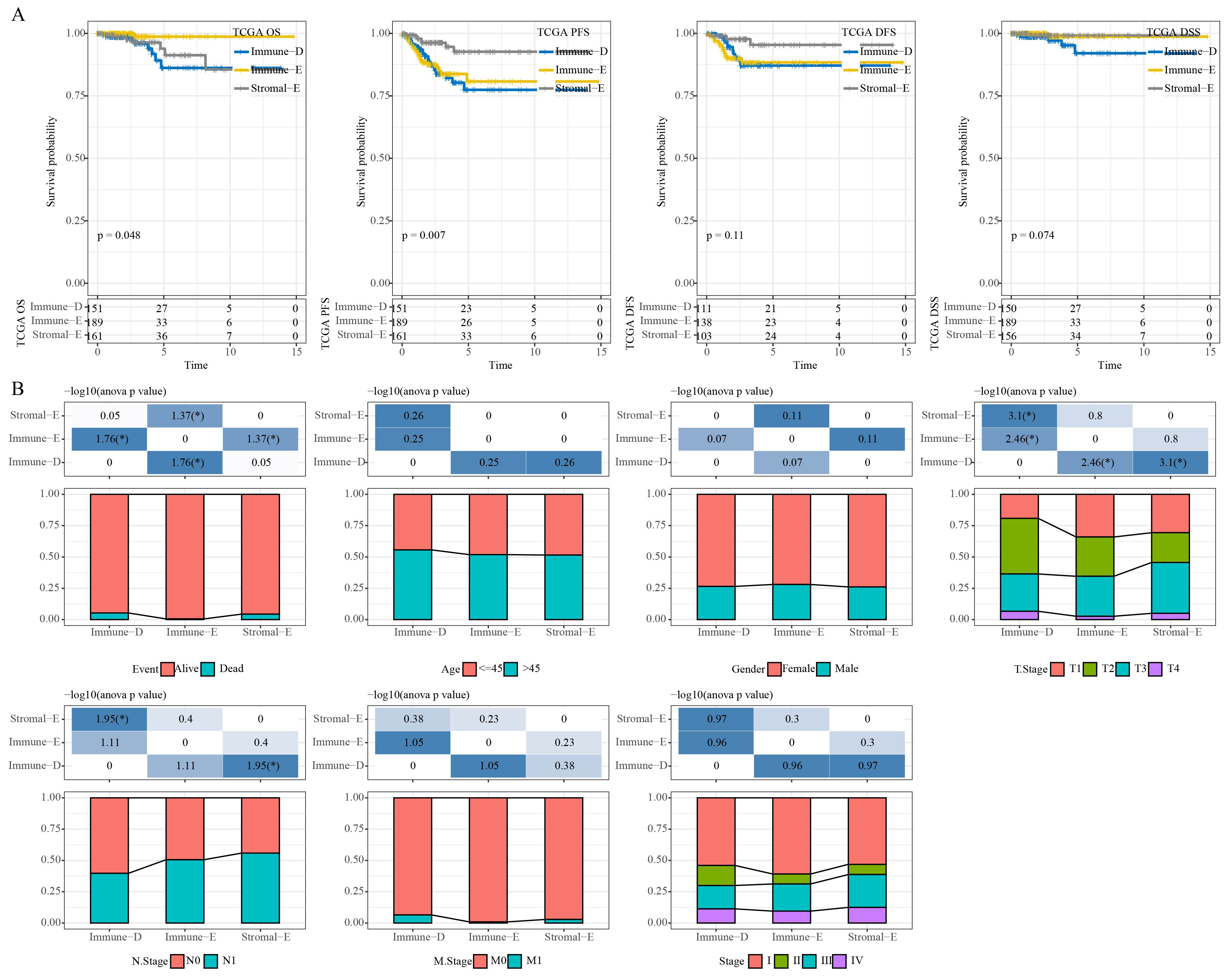

Supplement: Supplementary file 1 [file Image1.JPEG]

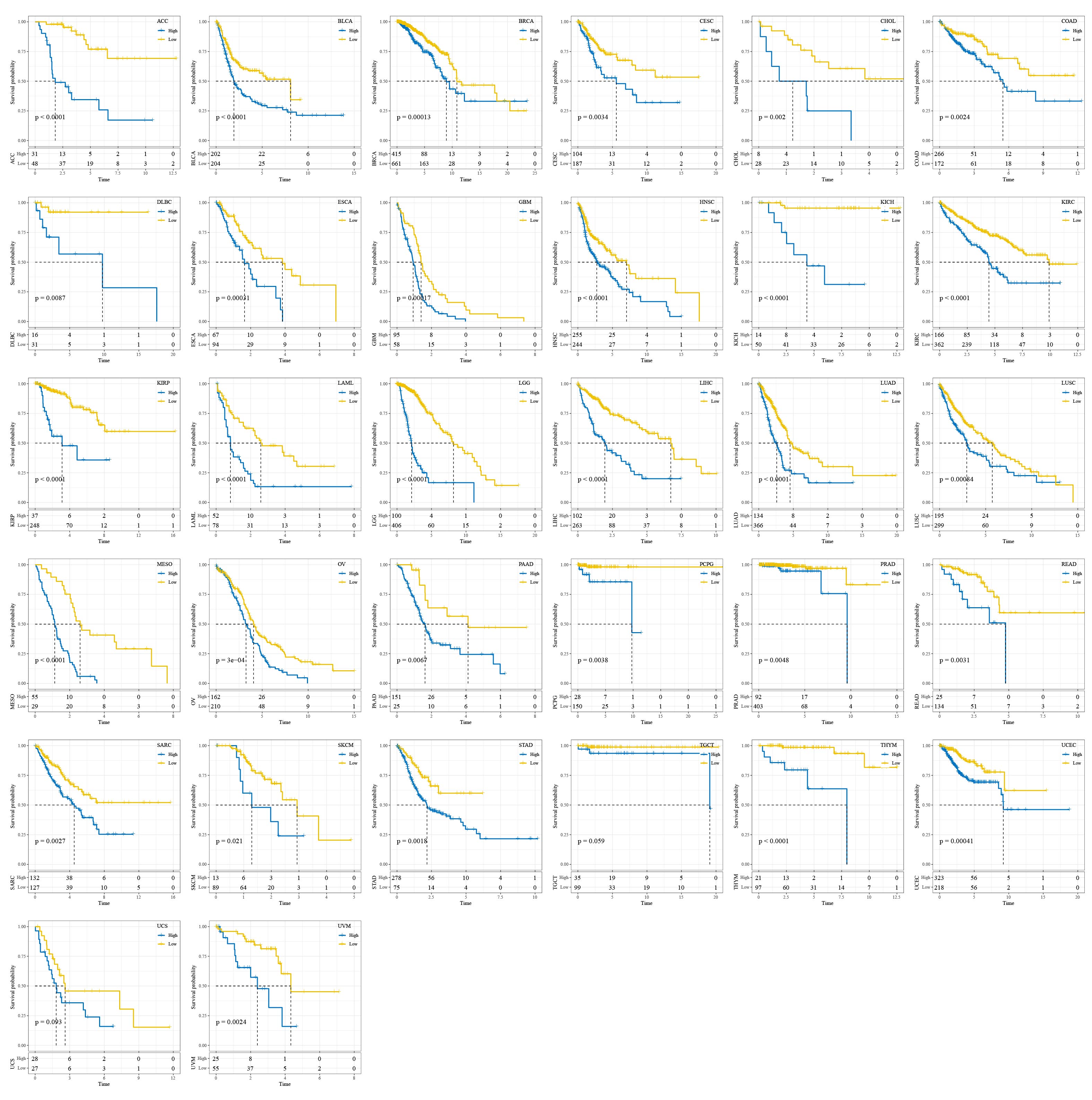

Supplement: Supplementary file 2 [file Image2.JPEG]
