# Supplementary material for: Identification of novel immune-related molecular subtypes and a prognosis model to predict thyroid cancer prognosis and drug resistance
Source: Front Pharmacol. 2023 Mar 30;14:1130399. doi: 10.3389/fphar.2023.1130399 (PMC10098004; doi:10.3389/fphar.2023.1130399)
Supplement: Supplementary file 3 [file DataSheet1.docx]

Supplementary figure legends

Figure S1

Clinical features analysis among 3 molecular subtypes. A: KM survival curve of 3 molecular subtypes. B: Clinical features analysis among 3 molecular subtypes.

Figure S2

KM survival curve of IMScore in 32 kinds cancers.
